# Effects of Human Rights Sensitivity of case managers on the working relationships with people suffering from mental illness mediated by empathy

**DOI:** 10.1192/j.eurpsy.2022.773

**Published:** 2022-09-01

**Authors:** M. Seo, Y.R. Kim

**Affiliations:** 1 Gyeongsang National University, Social Welfare, Jinju, Korea, Republic of; 2 Mokpo National University, Social Welfare, Mokpo, Korea, Republic of

**Keywords:** Human Rights Sensitivity, working relationships, Empathy

## Abstract

**Introduction:**

It is critical to provide not only mental health services but also welfare services that meet the socioeconomic needs of people suffering from mental illnesses in order for them to recover. Case managers in the public sector who provide socioeconomic support to the low-income class, in particular, play critical roles in early detection of untreated mentally ill people, linking them to the mental health system, and providing various supports for their community integration. Positive working relationships are required to fulfill these roles.

**Objectives:**

This study aims to analyze the effects of human rights sensitivity of case managers on the working relationships with the persons with mental illness mediated by empathy.

**Methods:**

We evaluated overall human rights sensitivity, level of empathy(cognitive, affective, behavioral aspects) and working relationships with the mentally ill of 291 public sector case managers(Mean age = 40.52, SD=7.96, female 78.2%, male 21.8%).

**Results:**

In research model analysis, the goodness-of-fit was evaluated to verify the effect of overall human rights sensitivity on the working relationships with the persons with mental illness mediated by empathy. Most of indices showed sufficient goodness-of-fit. In other words, the higher overall human rights sensitivity is, the higher the level of empathy is, and this has a positive effect on the working relationships with persons with mental illness.

**Conclusions:**

To form positive working relationships with people suffering from mental illnesses, public sector case managers must be educated to increase their empathy by improving their overall human rights sensitivity.

**Disclosure:**

No significant relationships.

